# Methylation differences between assisted reproductive technology-conceived and naturally conceived children near *BRCA1* and *NBR2*

**DOI:** 10.1080/15592294.2025.2577188

**Published:** 2025-10-21

**Authors:** Yunsung Lee, Håkon Kristian Gjessing, Christian Magnus Page, Jon Bohlin, Robert Lyle, Per Magnus, Siri Eldevik Håberg

**Affiliations:** a Centre for Fertility and Health, Norwegian Institute of Public Health, Oslo, Norway; b Department of Global Public Health and Primary Care, University of Bergen, Bergen, Norway; c Department of Physical Health and Ageing, Division of Public Health and Prevention, Norwegian Institute of Public Health, Oslo, Norway; d Department of Method Development and Analytics, Section for Modeling and Bioinformatics, Norwegian Institute of Public Health, Oslo, Norway; e Department of Medical Genetics, Oslo University Hospital, Oslo, Norway

**Keywords:** Assisted reproductive technology, DNA methylation, Norwegian mother, father, and child cohort study (MoBa), *BRCA1/NBR2*

## Abstract

Recent studies have shown that newborns conceived using assisted reproductive technology (ART) exhibited significantly different DNA methylation (DNAm) profiles at birth compared to those conceived naturally. Of note was the observation of increased DNAm at the promoter region of *BRCA1/NBR2* in ART-conceived newborns. However, it remains unclear if these DNAm differences persist after birth. Using the Norwegian Mother, Father, and Child Cohort Study (MoBa), a large-scale population-based pregnancy cohort with extensive longitudinal data collected through biological samples and questionnaires, we generated longitudinal DNAm data for 105 ART-conceived and 250 naturally conceived children at birth and at ages 3–22 years. DNAm differences in the *BRCA1*/*NBR2* promoter between ART- and naturally conceived children, at birth and postnatally, were tested using linear mixed model with adjustment for maternal and newborn covariates. While ART-conceived children showed subtle hypermethylation at birth and postnatally, the differences diminished over time and did not remain statistically significant after multiple testing correction. Our findings suggest that subtle hypermethylation at the *BRCA1/NBR2* promoter in ART-conceived children may represent an ART-associated epigenetic signature, although further studies in larger populations are needed to clarify its persistence and significance.

## Introduction

A recent publication by Håberg, Page [1] presented the largest epigenome-wide association study of assisted reproductive technology (ART) to date, identifying 607 CpG sites that were differentially methylated in cord blood between newborns conceived using assisted reproductive technology (ART) and those conceived naturally. Among these differentially methylated loci, multiple CpGs in the bidirectional promoter region of *BRCA1/NBR2* were found to be hypermethylated in ART-conceived newborns. Since *BRCA1* is a well-established susceptibility gene for early-onset breast cancer and ovarian tumors [2,3], this finding may have important health implications in later life. Aberrant DNA methylation (DNAm) in the promoter region of this gene may alter gene expression patterns [4,5], and promoter methylation of this gene in peripheral blood has been associated with an increased risk of sporadic breast cancer [6,7] and ovarian cancer [8].

Despite these findings, it remains unknown whether the observed hypermethylation of *BRCA1/NBR2* in ART-conceived children persists beyond birth or undergoes temporal changes during childhood as studies with longitudinal follow-up of DNAm changes are scarce. Yeung, Mendola [9] reported one differentially methylated locus (cg04061372 hg19 17:40,805,777) in ART-conceived (*n* = 23) and naturally conceived children (*n* = 93) at age 8–10 years, while reporting a null finding for *BRCA1* promoter methylation. Similarly, Novakovic, Lewis [10] conducted an epigenome-wide association study of the mode of conception (ART vs natural) in adults aged 22–35 years (*n* = 233) but did not report any differentially methylated CpGs at *BRCA1*/*NBR2*.

To examine whether DNAm differences in the *BRCA1* and *NBR2* bidirectional promoter persisted beyond birth, we used a longitudinal design with repeated DNAm measurements, with one time point at birth and an additional time point between the age of 3 and 22 years, in the same children.

## Methods

### Study population

MoBa is a nationwide pregnancy cohort in which approximately 95,000 mothers, 75,000 fathers, and 114,000 children were recruited from 1999 to 2008 across Norway [11]. The participation rate of invited pregnant women was 41%. The participants completed a series of questionnaires during pregnancy and after childbirth and are still followed up. Peripheral whole-blood samples were collected from mothers and fathers around the 17^th^ week of gestation and from the mothers (whole-blood) and newborn children (cord blood) at delivery [12,13]. Various sub-studies in MoBa have collected peripheral blood samples, from different subsets of children between the age of 3 and 22 years [14,15].

For this study, we selected 119 ART-conceived and 250 naturally conceived control children matched on age, sex, and on the sub-study providing follow-up samples, with available cord blood at birth and peripheral blood at later age in the MoBa biobank. Among the ART-conceived children, there were 14 twin pairs. In contrast, among the naturally conceived children, there were no twin pairs. To ensure independence among children, we randomly selected one child from each twin pair in the ART group, resulting in 105 ART-conceived and 250 naturally conceived children for analyses (Table 1).

**Table 1.** Descriptive statistics.

	Naturally- conceived (*n* = 250)		ART- conceived (*n* = 105)	
**Repeated measure of DNAm^1^**				
2 time points	243	(97.2%)	80	(76.2%)
1 time point	7	(2.8%)	25	(23.8%)
NA	0	(0%)	0	(0%)
**Child’s sex**				
Male	102	(40.8%)	46	(43.8%)
Female	148	(59.2%)	59	(56.2%)
NA	0	(0%)	0	(0%)
**Type of births^2^**				
Singleton	248	(99.2%)	81	(77.1%)
Multiple (Twin)	2	(0.8%)	24	(22.9%)
NA	0	(0%)	0	(0%)
**Maternal smoking during pregnancy**				
No	191	(76.4%)	91	(86.7%)
Sometimes	47	(18.8%)	9	(8.6%)
Daily	12	(4.8%)	5	(4.8%)
NA	0	(0%)	0	(0%)
**Maternal BMI before pregnancy**				
Underweight	7	(2.8%)	4	(3.8%)
Normal	172	(68.8%)	69	(65.7%)
Overweight	41	(16.4%)	22	(21%)
Obese	16	(6.4%)	7	(6.7%)
NA	14	(5.6%)	3	(2.9%)
**Maternal parity**				
0	125	(50%)	71	(67.6%)
1	82	(32.8%)	27	(25.7%)
2	36	(14.4%)	7	(6.7%)
3	5	(2%)	0	(0%)
4	2	(0.8%)	0	(0%)
NA	0	(0%)	0	(0%)
**Maternal age (in years)**				
< 20	1	(0.4%)	0	(0%)
[20, 25)	15	(6%)	0	(0%)
[25, 30)	75	(30%)	14	(13.3%)
[30, 35)	95	(38%)	42	(40%)
≥35	64	(25.6%)	49	(46.7%)
NA	0	(0%)	0	(0%)

^a^We measured DNAm levels at birth using cord blood samples and after birth (at age 3–22 years) using peripheral blood samples. Two time points were referred to as both at and after birth, while one time point was referred to as either of them.

^b^This item shows the number of children from singleton or multiple births. Prior to generating this table, we randomly selected one child from each twin pair. As a result, in this table, the counts do not include both members of any twin ‘pair’ – each pair contributes only one child.

### ART and covariates

ART procedures, including in vitro fertilization, intracytoplasmic sperm injection, and fresh or frozen-thawed embryo transfer, are reported by fertility clinics to the Medical Birth Registry of Norway (MBRN) which records all births nationwide. Using personal identification numbers, we could link the relevant information on ART procedures from MBRN to the MoBa children. Maternal smoking during pregnancy and body mass index (BMI) before pregnancy were obtained from the MoBa questionnaires, while parity, child’s sex, and child’s age were sourced from MBRN.

### Measurement of DNA methylation

We retrieved DNA extracts from the selected children, collected at birth (cord blood samples) and after birth (at age 3–22 years, peripheral blood samples) that were stored at the MoBa biobank [12]. These samples were shipped to the Life & Brain Centre of the University of Bonn (Bonn, Germany) for generation of DNAm data using the Illumina Infinium MethylationEPIC v2.0 array.

Bisulphite conversion was performed using the EZ-96 DNA Methylation-Lightning MagPrep kit (Zymo Research, Irvine, California, USA). Raw iDat files were processed with the RnBeads v.2.21.3 R package in two batches, with random allocation to each batch. Sample-level quality control (QC) followed the RnBeads defaults with explicit thresholds: we visually inspected the fluorescence intensities from the control probes for all samples and predicted sex from X/Y probe intensities was compared with sex registered on MBRN; no mismatches were observed. To remove unreliable measurements, we applied the ‘greedycut’ algorithm with a detection P of 0.01 at both the probe and sample levels. Additionally, at the probe level, we excluded probes with low bead count (minimum coverage set to 5). The normal-exponential using out-of-band probes by SeSAMe [16] was used for background correction, and the beta-mixture quantile normalization [17] was used for normalization of Type I and II probe intensities. The QCed DNAm data included 869,783 autosomal CpGs, 22,618 X-chromosome CpGs, and 131 Y-chromosome CpGs.

From the QCed DNAm data, the cell-type composition was estimated using the UniLIFE reference panel [18] and EpiDISH *R* package [19], which is well suitable for longitudinal design including cord and peripheral blood samples. For longitudinal consistency, we applied the cord-blood reference (including nRBC) at birth and the adult peripheral-blood reference postnatally. Statistical analyses were performed on M-values [20].

### Statistical analyses

First, we conducted association tests between the use of ART and DNAm in *BRCA1*/*NBR2* at birth using DNAm data from the cord blood samples. We then performed the same association tests after birth using the data from the peripheral blood samples. For outlier removal, in each dataset, we identified and excluded children whose M-values fell outside the range: median (M-value) ± 5 × median absolute deviation. We applied a linear mixed model to regress each CpG on fixed effects for the use of ART, maternal age, smoking, BMI, parity, child’s sex, child’s age (only applicable to DNAm after birth), multiple pregnancy, and a random effect for the 96-well plate. As a sensitivity analysis, we included cell-type composition as additional covariates in the model. We also performed another sensitivity analysis by excluding children from multiple births.

Second, we modelled the within-individual change in DNAm according to the use of ART. To account for sex, age (only applicable to DNAm data after birth), and plate/batch effect, we applied a linear mixed model to regress each CpG on these variables separately in DNAm data at birth and those after birth. The residuals from these regressions were then standardized within each dataset to ensure comparability. Next, we computed the difference in standardized residuals between birth and later age for each CpG site, reflecting changes in DNAm over time. This difference was then used as the outcome variable in an ordinary linear model, where we examined its association with ART exposure while adjusting for maternal age, smoking status, BMI, and parity.

All the analyses were performed in R 4.1.2., and the linear mixed model was implemented using the lme function from the nlme R package. The two-sided Wald test was used to assess statistical significance. Relevant R code can be downloaded from https://github.com/yunsunglee-dev/BRCA-NBR2-methylation-in-ART-conceived-children.git.

## Results

This study included a total of 250 naturally conceived children and 105 ART-conceived children (Table 1). For the 250 naturally conceived ones, we measured 247 individuals’ DNAm levels at birth in cord blood samples and 246 individuals’ DNAm after birth (at age 3–22 years) in peripheral blood samples (Figure S1, Supplemental File 1). For the 105 ART-conceived children, 81 individuals had available DNAm at birth, and 104 individuals had available DNAm after birth.

The distributions of the after-birth samples’ age were as follows: 81 individuals at age 0–5 years (naturally conceived *n* = 53 and ART *n* = 28), 21 individuals at 5–10 years (naturally conceived *n* = 19 and ART *n* = 2), 198 individuals at 10–15 years (naturally conceived *n* = 137 and ART *n* = 61), 43 individuals at 15–20 years (naturally conceived *n* = 33 and ART *n* = 10), and 7 individuals at 20–25 years (naturally conceived *n* = 4 and ART *n* = 3). These are visualized in Figure S1, Supplemental File 1.

The mothers of the ART-conceived children (33.8 years old) were, on average, older than those of the naturally conceived children (31.3 years old). The difference in maternal age was statistically significant (P-value = 8.5E-07).

**Figure 1.**
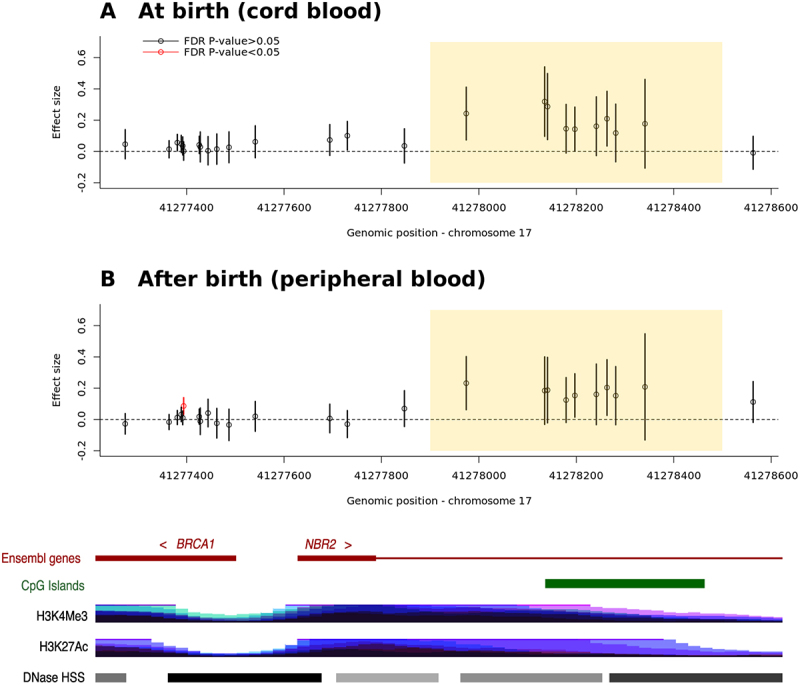
Dnam differences at *BRCA1* and *NBR2* in ART- and naturally conceived children at and after birth.

We first examined the DNAm-ART association at birth, focusing on 25 CpGs located within the *BRCA1*/*NBR2* promoter (hg19 17:41,277,059–41,278,712) previously reported by Håberg, Page [1]. As expected, a group of CpGs at the *BRCA1/NBR2* promoter showed moderate hypermethylation in ART-conceived newborns as compared to naturally conceived ones (highlighted in yellow in Figure 1(A)). Although these DNAm-ART associations did not reach statistically significance after multiple testing correction, e.g., false discovery rate (FDR) <5%, the overall trend was similar to the previous findings (Figure 7(C) in Håberg, Page [1]).

The DNAm differences between ART- and naturally conceived children are presented as effect sizes on the y-axis. The effect sizes are shown (A) at birth (cord blood) and (B) after birth (peripheral blood, at age 3–22 years). Each dot represents the effect size of a DNAm-ART association at a CpG site, with black indicating FDR-adjusted p-value >0.05 and red indicating FDR-adjusted p-value <0.05. The yellow-highlighted region marks the genomic region where Håberg, Page [1] found hypermethylation in ART-conceived children compared to naturally conceived ones.

Next, we investigated whether these DNAm-ART associations identified above, persisted after birth by analyzing DNAm data from the same individuals at the age of 3–22 years. Again, we observed a similar trend with slight hypermethylation at the same CpG sites in ART-conceived children, although it did not reach statistical significance (Figure 1(B) and Table S1).

As a sensitivity analysis, we adjusted for estimated cell-type composition, which did not alter the results (Figure S2, Supplemental File 1). We also excluded the children from multiple births and performed the same analysis. The results remained unchanged (Figure S3, Supplemental File 1).

To assess whether DNAm-ART associations at birth differed from those after birth, we leveraged the longitudinal design of this study with repeated DNAm measurement in same individuals. Specifically, we calculated within-individual differences in DNAm level at each CpG between birth and later age and examined their associations with ART. We found no statistically significant associations between ART and the change in DNAm levels over time, indicating that there is no clear evidence that hypermethylation in ART-conceived children disappears after birth (Table 2).

**Table 2.** Association between ART and within-child changes in DNAm at *BRCA1* and *NBR2* CpG sites.

CpG site	CHR^1^	Position^1^	Regulatory Feature Group^2^	Relation to UCSC CpG Island^2^	UCSC RefGene Name^2^			
						Beta coefficient^3^ (95% confidence interval)	Raw P-value	FDR-corrected P-value^4^
cg08386886	17	41,277,274	Promoter Associated	N Shore	*BRCA1;NBR2*	−0.067 (−0.184, 0.049)	0.258	0.785
cg24806953	17	41,277,364	Promoter Associated	N Shore	*BRCA1;NBR2*	−0.016 (−0.085, 0.054)	0.661	0.787
cg20187250	17	41,277,381	Promoter Associated	N Shore	*BRCA1;NBR2*	−0.038 (−0.108, 0.032)	0.287	0.785
cg15419295	17	41,277,389	Promoter Associated	N Shore	*BRCA1;NBR2*	−0.02 (−0.094, 0.053)	0.587	0.787
cg16963062	17	41,277,392	Promoter Associated	N Shore	*BRCA1;NBR2*	−0.019 (−0.092, 0.054)	0.609	0.787
cg16630982	17	41,277,394	Promoter Associated	N Shore	*BRCA1;NBR2*	0.088 (0.012, 0.165)	0.025	0.488
cg21253966	17	41,277,426	Promoter Associated	N Shore	*BRCA1;NBR2*	−0.023 (−0.096, 0.051)	0.547	0.787
cg04110421	17	41,277,428	Promoter Associated	N Shore	*BRCA1;NBR2*	−0.056 (−0.187, 0.076)	0.408	0.787
cg04658354	17	41,277,444	Promoter Associated	N Shore	*BRCA1;NBR2*	0.065 (−0.064, 0.194)	0.326	0.785
cg17301289	17	41,277,462	Promoter Associated	N Shore	*BRCA1;NBR2*	0.002 (−0.136, 0.14)	0.976	0.976
cg09441966	17	41,277,487	Promoter Associated	N Shore	*BRCA1;NBR2*	−0.045 (−0.188, 0.099)	0.544	0.787
cg26891576	17	41,277,541	Promoter Associated	N Shore	*BRCA1;NBR2*	−0.031 (−0.171, 0.108)	0.660	0.787
cg10893007	17	41,277,694	Promoter Associated	N Shore	*BRCA1;NBR2*	−0.079 (−0.207, 0.049)	0.229	0.785
cg12182452	17	41,277,730	Promoter Associated	N Shore	*BRCA1;NBR2*	−0.088 (−0.213, 0.037)	0.170	0.785
cg09831010	17	41,277,847	Promoter Associated	N Shore	*BRCA1*	0.073 (−0.063, 0.209)	0.294	0.785
cg25067162	17	41,277,974	Promoter Associated	N Shore	*BRCA1*	−0.02 (−0.105, 0.064)	0.636	0.787
cg26276233	17	41,278,135		Island	*BRCA1*	−0.119 (−0.231, −0.006)	0.039	0.488
cg06001716	17	41,278,141		Island	*BRCA1*	−0.114 (−0.241, 0.013)	0.080	0.664
cg02286533	17	41,278,179		Island	*BRCA1*	−0.034 (−0.114, 0.047)	0.415	0.787
cg14947218	17	41,278,197		Island	*BRCA1*	0.009 (−0.06, 0.079)	0.795	0.903
cg16006004	17	41,278,241		Island	*BRCA1*	−0.007 (−0.113, 0.099)	0.899	0.976
cg18372208	17	41,278,263		Island	*BRCA1*	−0.052 (−0.161, 0.056)	0.345	0.785
cg14687474	17	41,278,281		Island	*BRCA1*	0.003 (−0.092, 0.098)	0.950	0.976
cg25288140	17	41,278,341		Island	*BRCA1*	0.043 (−0.138, 0.223)	0.643	0.787
cg27581762	17	41,278,563		S Shore	*BRCA1*	0.1 (−0.041, 0.241)	0.165	0.785

^a^Based on Genome Reference Consortium Human Build 37.

^b^Retrieved from the Illumina Infinium MethylationEPIC v2.0 manifest file.

^c^Derived from the regression of within-child difference in DNAm between at and after birth on ART and covariates.

^d^The Benjamini-Hochberg procedure was applied.

We visually inspected scatter plots of the DNAm levels at CpGs against child’s age (Figure S4, Supplemental File 1). Among the CpGs located in the genomic region where Håberg, Page [1] found hypermethylation in ART-conceived children, we observed a tendency of differences in M-values between ART-conceived and naturally conceived children diminishing as child’s age increased. However, this interpretation was based on visual inspection, not based on statistical test such as the within-child difference model mentioned above.

## Discussion

We found subtle hypermethylation at the *BRCA1*/*NBR2* promoter in ART-conceived children compared to naturally conceived children both at birth and at later ages up to 22 years. Hypermethylation in the promoter region was observed at the same CpGs at birth and later ages. Though hypermethylation at later ages was slightly reduced compared to that at birth, the difference in the intensity of hypermethylation between at birth and at later ages was not statistically significant.

Several previous studies explored epigenetic differences between ART-conceived and naturally conceived children using different types of tissues, e.g., cord blood, placenta, and peripheral blood [21]. Among these studies, only one study by Håberg, Page [1] identified hypermethylation in ART-conceived children at *BRCA1/NBR2*. Although this finding has not yet been replicated, we consider it unlikely to be false positive, given the study’s large sample size (approximately 1,000 ART and 1,000 naturally conceived), control for parental DNAm, stringent multiple testing threshold (FDR < 1%), and the observation of increased DNAm at 10 consecutive CpGs. Two other studies that analyzed peripheral blood samples from children or adolescents, Yeung, Mendola [9] and Penova-Veselinovic, Melton [22], did not report statistically significant hypermethylation of *BRCA1*/*NBR2* in ART-conceived individuals.

The *BRCA1* promoter is normally unmethylated in peripheral blood [23], resulting in an unsilenced promoter and intact tumor-suppressor activity. Rarely, however, constitutional *BRCA1* promoter methylation (occurring in non-tumor tissues such as peripheral blood) has been linked to increased risk of early onset breast cancer and ovarian cancer [7,24,25]. Although we found no strong evidence of the *BRCA1*/*NBR2* promoter hypermethylation in ART-conceived children at the ages studied, it is still plausible that ART-conceived children show hypermethylation in their later life. McCartney, Zhang [26] reported two promoter CpGs, i.e., cg08386886 and cg26891576, showing modest correlation with chronological age, and peripheral blood methylation at these two CpGs together with their neighboring CpGs have been associated with triple-negative breast cancers and high-grade serous ovarian cancers (see Lonning, Nikolaienko [24]; CpG17 and CpG32 listed in eTable 1).

Furthermore, a recent study by Ruiz-Arenas, Hernandez-Ferrer [4] reported that increased methylation at cg25067162, located ~150 bp proximal to the CpG island, significantly reduced the expression levels of *NBR2* (TC17000547.hg.1, fold change (FC) = 0.93, P-value = 3.61E-42) and TC17002214.hg.1 (FC = 0.92, P-value = 2.93E-43) in children’s peripheral blood. *NBR2*, a long non-coding RNA, has been suggested to be a regulatory element in cancer biology [27]. Taken together, increased DNAm in this region may have functional consequences.

We excluded outliers to secure the stability of linear mixed models. In such models, coefficient estimates – here, the differences in methylation between naturally-conceived and ART-conceived children – can be largely influenced by a handful of ‘technical’ outliers. Nevertheless, we do not rule out the possibility that outliers were ‘biologically’ driven [28,29]. For example, Ghosh, Mainigi [30] reported that children with low birthweight showed more methylation outliers than those with high birthweight, and among the children with low birthweight, those conceived in-vitro had more outliers in methylation than those conceived in-vivo. However, we did not observe a similar pattern in our data. In the peripheral blood (after-birth) methylation data, cg04110421 had the highest number of outliers (*n* = 11, all positive), with seven from naturally conceived children and four from ART-conceived children. Given the sample sizes (246 naturally conceived and 104 ART-conceived), this distribution of outliers was within expectation. Similar trends were observed across other CpGs, suggesting that outliers were unlikely to influence our findings.

This study has several limitations. First, the sample size limited our ability to detect small DNAm differences with statistical significance. According to our post-hoc power analysis of the cord blood DNAm data, 4.5-folds increase in sample size (1,112 naturally conceived and 365 ART-conceived) would be required to achieve 80% power in detecting the methylation difference at cg02286533. This estimate was based on a Monte-Carlo simulation with 10,000 iterations that assumed a true methylation difference of 0.13 in M-values (derived from Håberg, Page [1]), a standard deviation of 0.49 in both groups’ M-values (also derived from Håberg, Page [1]). To simulate slight heavy tails, a student t distribution with 10 degrees of freedom was used. In mathematical terms, the simulation model was $M_{i}=1.2+0.13*X_{\mathrm{naturalvsART},i}+\varepsilon_{i}$, where $\varepsilon_{i}∼t\left(\mathrm{df}=10\right)*0.49$. The statistical significance was defined conservatively as *p* < 0.05/25 = 0.002 (Bonferroni correction). For the peripheral blood DNAm data, we would have the confidence intervals and p-values included in Figure 1(B) reflect the statistical power as no prior study exists to inform us of key parameters for power calculation. Second, our study population consisted of individuals of Northern European ancestry, which may limit the generalizability of our findings to more diverse populations. Future studies with larger and more diverse cohorts are needed to validate and expand upon our results. Finally, this study did not expand its scope to the genome-wide level but focused on the *BRCA1/NBR2* region. This decision was made a priori, as the study was conceived as a hypothesis-driven evaluation of this locus based on its established clinical relevance to cancer susceptibility and prior ART-related methylation findings by Håberg, Page [1]. In addition, the limited availability of postnatal peripheral blood samples in MoBa would have substantially reduced the statistical power for a discovery-scale epigenome-wide analysis, increasing the risk of generating underpowered or misleading results.

In conclusion, our findings suggest that subtle and persistent hypermethylation at the *BRCA1/NBR2* promoter in ART-conceived children may reflect an epigenetic alteration with potential functional relevance, warranting further investigation in larger and more diverse populations.

## Supplementary Material

Supplemental_File1.docx

S_Table1.xlsx

## Data Availability

Access to the genetic datasets can be obtained by applying to the Norwegian Institute of Public Health (NIPH; http://www.fhi.no/en/). Restrictions may apply regarding the availability of these data, which were originally used under specific approvals for the current study and are therefore not publicly available. Access can only be given after approval by REK under the provision that the applications are consistent with the consent provided. An application form can be found on the NIPH website at https://www.fhi.no/en/studies/moba/. Specific questions regarding access to data in this study can also be directed to Dr. Siri E. Håberg (sirieldevik.haberg@fhi.no). Table S1 can be downloaded via the following link: https://docs.google.com/spreadsheets/d/12XlhVoRyz5qna5i9xDUpWVlqO-JG2ebF/edit?usp=sharing&ouid=117867346092273626899&rtpof=true&sd=true.

## ABBREVIATIONS

MoBa: Norwegian Mother, Father, and Child Cohort Study
DNAm: DNA methylation

## References

[1] Håberg SE, Page CM, Lee Y, et al. Dna methylation in newborns conceived by assisted reproductive technology. Nat Commun. 2022;13(1):1896. doi: 10.1038/s41467-022-29540-w 35393427 PMC8989983

[2] Miki Y, Swensen J, Shattuck-Eidens D, et al. A strong candidate for the breast and ovarian cancer susceptibility gene BRCA1. Science. 1994;266(5182):66–9. doi: 10.1126/science.7545954 7545954

[3] Esteller M, Silva JM, Dominguez G, et al. Promoter hypermethylation and BRCA1 inactivation in sporadic breast and ovarian tumors. J Natl Cancer Inst. 2000;92(7):564–569. 10749912 10.1093/jnci/92.7.564

[4] Ruiz-Arenas C, Hernandez-Ferrer C, Vives-Usano M, et al. Identification of autosomal cis expression quantitative trait methylation (cis eQTMs) in children’s blood. Elife. 2022;11:11. doi: 10.7554/eLife.65310 PMC8933004 35302492

[5] Kumar M, Sahu RK, Goyal A, et al. Brca1 promoter methylation and expression - associations with ER+, PR+ and HER2+ subtypes of breast carcinoma. Asian Pac J Cancer Prev. 2017;18(12):3293–3299. doi: 10.22034/APJCP.2017.18.12.3293 29286222 PMC5980886

[6] Bosviel R, Garcia S, Lavediaux G, et al. Brca1 promoter methylation in peripheral blood DNA was identified in sporadic breast cancer and controls. Cancer Epidemiol. 2012;36(3):e177–82. doi: 10.1016/j.canep.2012.02.001 22402307

[7] Iwamoto T, Yamamoto N, Taguchi T, et al. Brca1 promoter methylation in peripheral blood cells is associated with increased risk of breast cancer with BRCA1 promoter methylation. Breast Cancer Res Treat. 2011;129(1):69–77. doi: 10.1007/s10549-010-1188-1 20882403

[8] Lonning PE, Berge EO, Bjornslett M, et al. White blood cell BRCA1 promoter methylation status and ovarian cancer risk. Ann Intern Med. 2018;168(5):326–334. doi: 10.7326/M17-0101 29335712

[9] Yeung EH, Mendola P, Sundaram R, et al. Conception by fertility treatment and offspring deoxyribonucleic acid methylation. Fertil Steril. 2021;116(2):493–504. doi: 10.1016/j.fertnstert.2021.03.011 33823999 PMC8349775

[10] Novakovic B, Lewis S, Halliday J, et al. Assisted reproductive technologies are associated with limited epigenetic variation at birth that largely resolves by adulthood. Nat Commun. 2019;10(1):3922. doi: 10.1038/s41467-019-11929-9 31477727 PMC6718382

[11] Magnus P, Birke C, Vejrup K, et al. Cohort profile update: the Norwegian Mother and Child Cohort Study (MoBa). Int J Epidemiol. 2016;45(2):382–388. doi: 10.1093/ije/dyw029 27063603

[12] Ronningen KS, Paltiel L, Meltzer HM, et al. The biobank of the Norwegian Mother and Child Cohort Study: a resource for the next 100 years. Eur J Epidemiol. 2006;21(8):619–625. doi: 10.1007/s10654-006-9041-x 17031521 PMC1820840

[13] Paltiel L, Anita H, Skjerden T, et al. The biobank of the Norwegian mother and child cohort study–present status. Nor Epidemiologi. 2014;24(1–2). doi: 10.5324/nje.v24i1-2.1755

[14] Nystad W, Magnus MC, Parr CL, et al. Causal pathways for asthma (CASPAR)–MoBa “a happy hunting ground”. Nor Epidemiologi. 2014;24(1–2). doi: 10.5324/nje.v24i1-2.1809

[15] Surén P, Schjølberg S, Øyen A-S, et al. The autism birth cohort (ABC): a study of autism spectrum disorders in MoBa. Nor Epidemiologi. 2014;24(1–2). doi: 10.5324/nje.v24i1-2.1757

[16] Zhou W, Triche TJ Jr., Laird PW, et al. Sesame: reducing artifactual detection of DNA methylation by Infinium BeadChips in genomic deletions. Nucleic Acids Res. 2018;46(20):e123. doi: 10.1093/nar/gky691 30085201 PMC6237738

[17] Teschendorff AE, Marabita F, Lechner M, et al. A beta-mixture quantile normalization method for correcting probe design bias in Illumina Infinium 450 k DNA methylation data. Bioinformatics. 2013;29(2):189–196. doi: 10.1093/bioinformatics/bts680 23175756 PMC3546795

[18] Guo X, Sulaiman M, Neumann A, et al. Unified high-resolution immune cell fraction estimation in blood tissue from birth to old age. bioRxiv. 2025:2025.02. 02.636167. 10.1186/s13073-025-01489-7 PMC12108007 40426256

[19] Teschendorff AE, Breeze CE, Zheng SC, et al. A comparison of reference-based algorithms for correcting cell-type heterogeneity in epigenome-wide association studies. BMC Bioinf. 2017;18(1):105. doi: 10.1186/s12859-017-1511-5 PMC5307731 28193155

[20] Du P, Zhang X, Huang CC, et al. Comparison of beta-value and M-value methods for quantifying methylation levels by microarray analysis. BMC Bioinf. 2010;11(1):587. doi: 10.1186/1471-2105-11-587 PMC3012676 21118553

[21] Schaub AM, Gonzalez TL, Dorfman AE, et al. A systematic review of genome-wide analyses of methylation changes associated with assisted reproductive technologies in various tissues. Fertil Steril. 2024;121(1):80–94. doi: 10.1016/j.fertnstert.2023.10.007 37827482 PMC11262788

[22] Penova-Veselinovic B, Melton PE, Huang RC, et al. DNA methylation patterns within whole blood of adolescents born from assisted reproductive technology are not different from adolescents born from natural conception. Hum Reprod. 2021;36(7):2035–2049. doi: 10.1093/humrep/deab078 33890633

[23] Snell C, Krypuy M, Wong EM, et al. Brca1 promoter methylation in peripheral blood DNA of mutation negative familial breast cancer patients with a BRCA1 tumour phenotype. Breast Cancer Res. 2008;10(1):R12. doi: 10.1186/bcr1858 18269736 PMC2374968

[24] Lonning PE, Nikolaienko O, Pan K, et al. Constitutional BRCA1 methylation and risk of incident triple-negative breast cancer and high-grade serous ovarian cancer. JAMA Oncol. 2022;8(11):1579–1587. doi: 10.1001/jamaoncol.2022.3846 36074460 PMC9459895

[25] Wong EM, Southey MC, Fox SB, et al. Constitutional methylation of the BRCA1 promoter is specifically associated with BRCA1 mutation-associated pathology in early-onset breast cancer. Cancer Prev Res (Phila). 2011;4(1):23–33. doi: 10.1158/1940-6207.CAPR-10-0212 20978112 PMC4030007

[26] McCartney DL, Zhang F, Hillary RF, et al. An epigenome-wide association study of sex-specific chronological ageing. Genome Med. 2019;12(1):1. doi: 10.1186/s13073-019-0693-z 31892350 PMC6938636

[27] Wang T, Li Z, Yan L, et al. Long non-coding RNA neighbor of BRCA1 gene 2: a crucial regulator in cancer biology. Front Oncol. 2021;11:783526. doi: 10.3389/fonc.2021.783526 34926299 PMC8674783

[28] Teschendorff AE, Gao Y, Jones A, et al. DNA methylation outliers in normal breast tissue identify field defects that are enriched in cancer. Nat Commun. 2016;7(1):10478. doi: 10.1038/ncomms10478 26823093 PMC4740178

[29] Teschendorff AE, Jones A, Widschwendter M. Stochastic epigenetic outliers can define field defects in cancer. BMC Bioinf. 2016;17(1):178. doi: 10.1186/s12859-016-1056-z PMC4840974 27103033

[30] Ghosh J, Mainigi M, Coutifaris C, et al. Outlier DNA methylation levels as an indicator of environmental exposure and risk of undesirable birth outcome. Hum Mol Genet. 2016;25(1):123–129. doi: 10.1093/hmg/ddv458 26566672 PMC4690496

