# Supplementary material for: Methylation differences between assisted reproductive technology-conceived and naturally conceived children near BRCA1 and NBR2
Source: Epigenetics. 2025 Oct 21;20(1):2577188. doi: 10.1080/15592294.2025.2577188 (PMC12548064; doi:10.1080/15592294.2025.2577188)
Supplement: Supplemental_File1.docx [file KEPI_A_2577188_SM7755.docx]

**Supplemental File 1**

### S-Figure 1. Data structure.


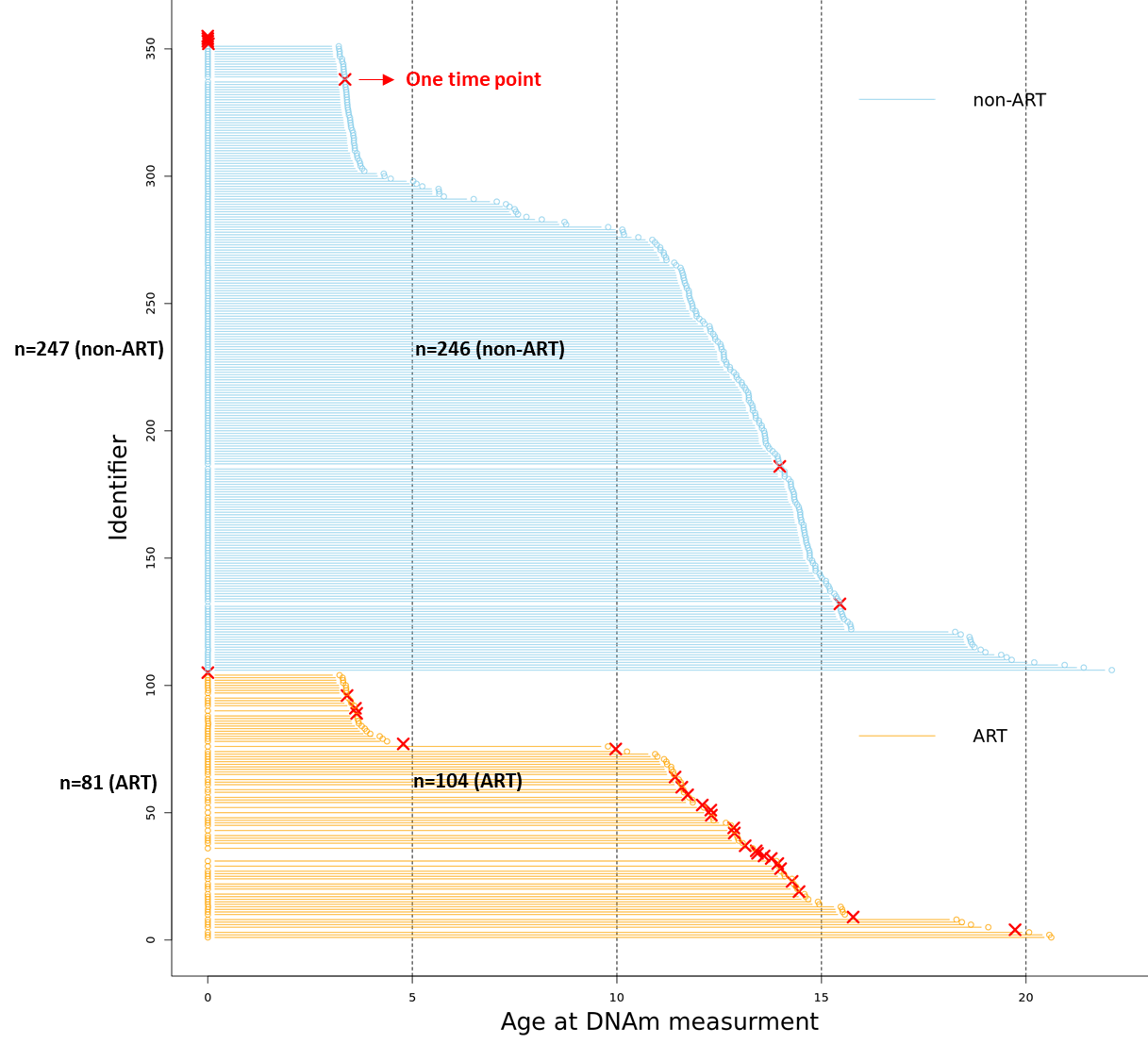


Each horizontal bar represents an individual with two DNAm measurements: one at birth and one after birth (the exact age at follow-up is shown on the X-axis). Individuals with only a single DNAm measurement are indicated by a red "X". The blue bars represent naturally-conceived children (non-ART), while the orange bars represent ART-conceived children.

### S-Figure 2. DNAm differences at BRCA1 and NBR2 in ART- and naturally-conceived children at and after birth after adjusting for cell type composition.


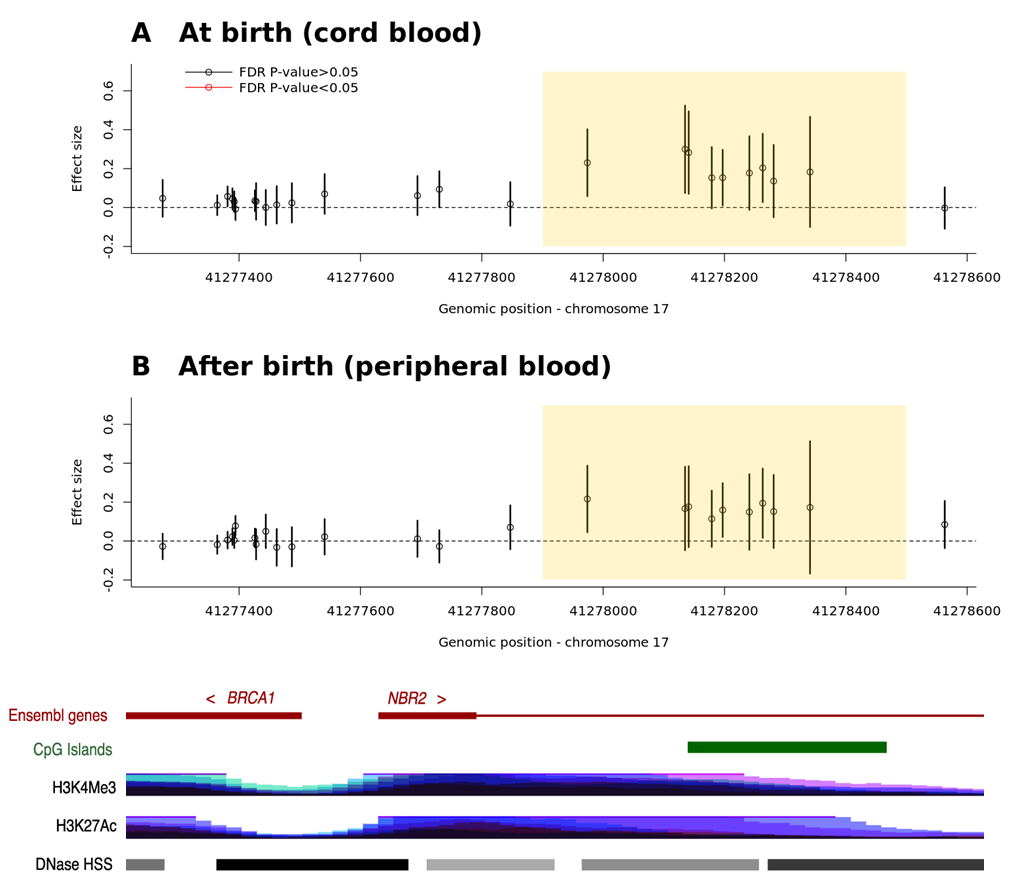


The DNAm differences between ART- and naturally conceived children are presented as effect sizes on the y-axis. The effect sizes are shown (A) at birth (cord blood) and (B) after birth (peripheral blood, at age 3 to 22 years). Each dot represents the effect size of an DNAm-ART association at a CpG site, with black indicating FDR-adjusted p-value>0.05 and red indicating FDR-adjusted p-value<0.05. The yellow-highlighted region marks the genomic region where Håberg, Page [1] found hypermethylation in ART-conceived children compared to naturally-conceived ones.

### S-Figure 3. DNAm differences at BRCA1 and NBR2 in ART- and naturally-conceived children at and after birth after excluding children from multiple births.


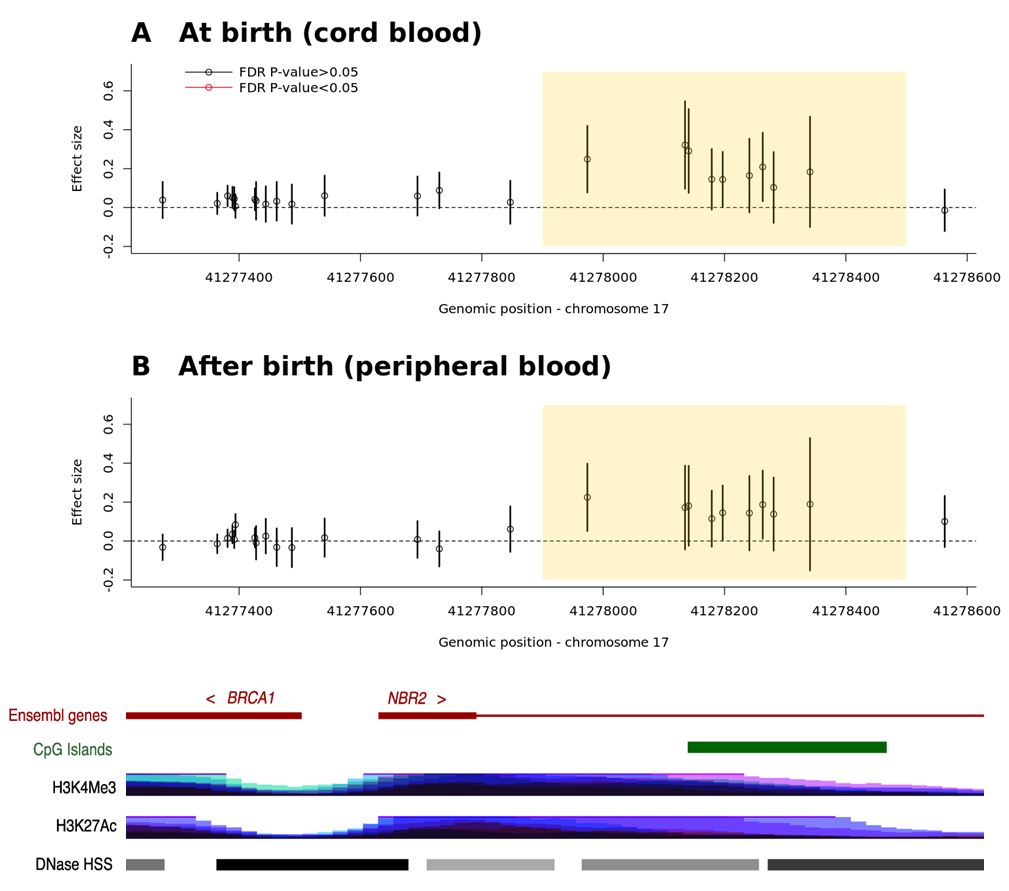


The DNAm differences between ART- and naturally conceived children are presented as effect sizes on the y-axis. The effect sizes are shown (A) at birth (cord blood) and (B) after birth (peripheral blood, at age 3 to 22 years). Each dot represents the effect size of an DNAm-ART association at a CpG site, with black indicating FDR-adjusted p-value>0.05 and red indicating FDR-adjusted p-value<0.05. The yellow-highlighted region marks the genomic region where Håberg, Page [1] found hypermethylation in ART-conceived children compared to naturally-conceived ones.

### S-Figure 4. Temporal change in DNAm level according to the mode of conception.


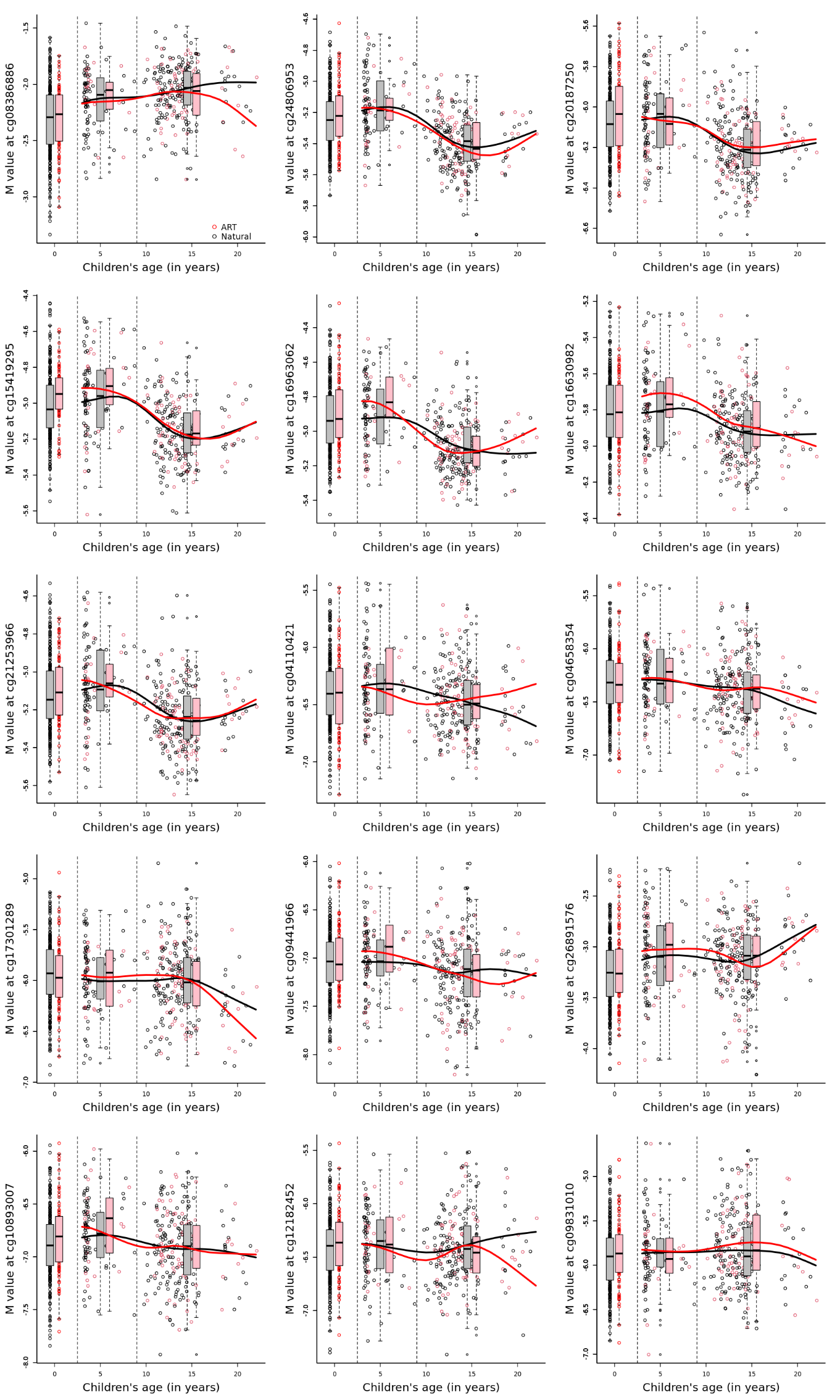


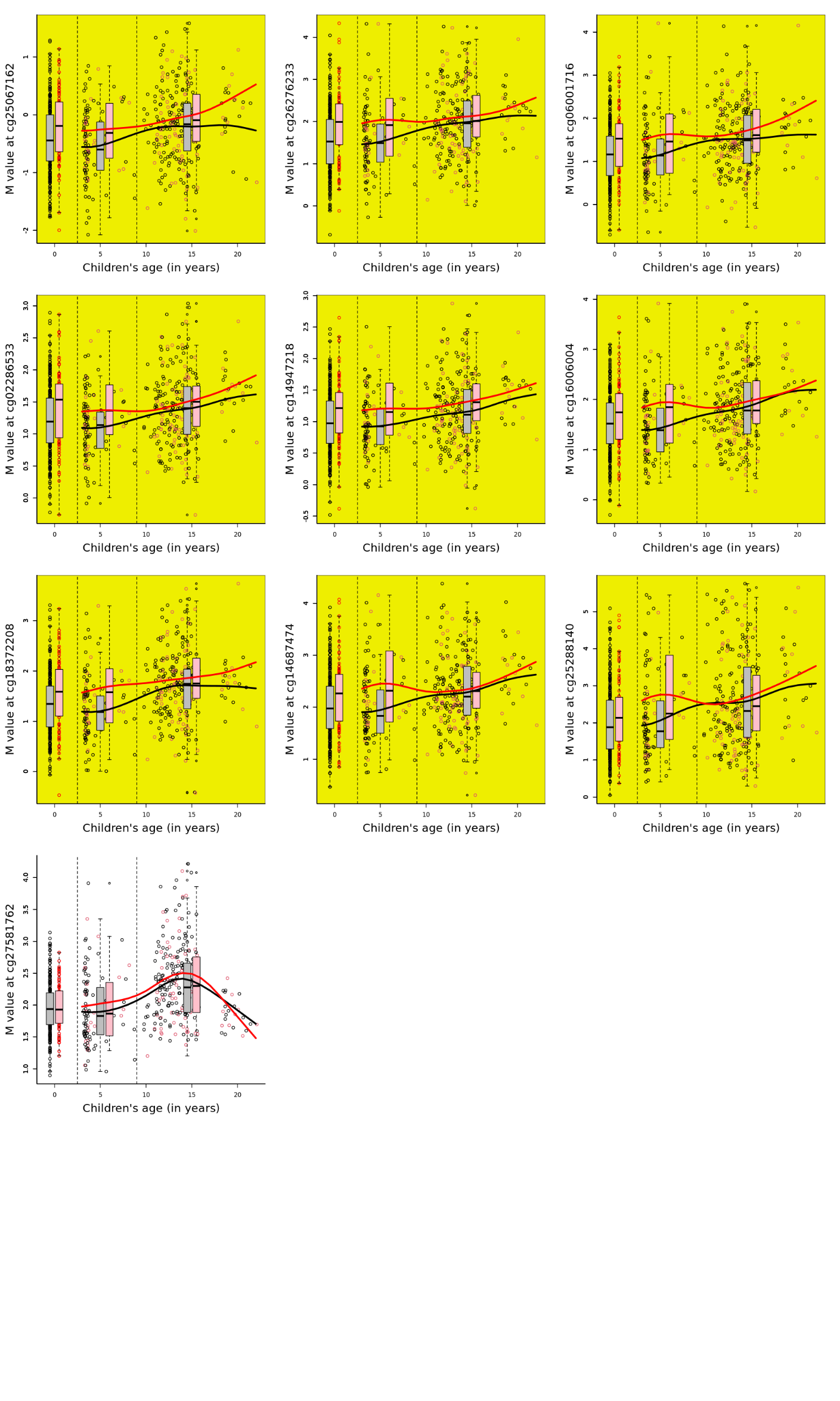


Each panel shows the relationship between M-values at a CpG and children’s age in years. The red dots represent ART-conceived children, while the black dots represent naturally conceived children. For better visibility, the M-values for the naturally conceived children at birth were shown slightly left from zero, and those for the ART-conceived children at birth were shown slightly right from zero. Their ages were all zero. The dotted vertical lines were located at age 2.5 and 9 years. Each plot includes two smoothing splines: one for ART-conceived children (red) and one for naturally conceived children (black), both fitted using peripheral blood sample data. The scatter plots for the CpGs the genomic region where Håberg, Page [1] found hypermethylation in ART-conceived children were colored in yellow.

**References**

[1] Håberg SE, Page CM, Lee Y, et al. DNA methylation in newborns conceived by assisted reproductive technology. Nature Communications. 2022;13(1):1896.
